# A Case of Metastatic Adamantinoma That Responded Well to Sunitinib

**DOI:** 10.1155/2016/5982313

**Published:** 2016-08-17

**Authors:** Andrew D. Liman, Agnes K. Liman, Jenna Shields, Becky Englert, Rashmikant Shah

**Affiliations:** ^1^Hematology and Oncology, VA Pittsburgh Healthcare System, University of Pittsburgh School of Medicine, Pittsburgh, PA 15240, USA; ^2^Pathology, VA Pittsburgh Healthcare System, University of Pittsburgh School of Medicine, Pittsburgh, PA 15240, USA; ^3^Pharmacy, VA Pittsburgh Healthcare System, Pittsburgh, PA 15240, USA; ^4^Hematology and Oncology, VA Pittsburgh Healthcare System, Pittsburgh, PA 15240, USA; ^5^Radiology, VA Pittsburgh Healthcare System, University of Pittsburgh School of Medicine, Pittsburgh, PA 15240, USA

## Abstract

Adamantinoma is a rare low-grade malignant bone tumor of epithelial origin. Metastatic adamantinoma has been reported to be resistant to chemotherapy. We report a case of metastatic adamantinoma to the lung, 10 years after the initial diagnosis of tibial mass. The patient received radiation therapy to the lung with partial response. A surveillance PET scan revealed progression of the lung mass and biopsy confirmed to be progressive residual metastatic adamantinoma. He received carboplatin and etoposide for 7 months and achieved a partial response. Four months later, PET scan showed disease progression. We started him on sunitinib, a multikinase inhibitor. He achieved a good partial response for 3 years. He died due to pneumonia at the age of 72.

## 1. Introduction

Adamantinoma is a low-grade, malignant bone tumor that is predominantly located in the midportion of the tibia [1, 2]. Adamantinoma constitutes 0.1% to 0.5% of all malignant bone tumors. The predilection is mainly involving the midshaft of the tibia which accounts for about 85% of all cases [1]. The initial symptoms of adamantinoma are often indolent and include swelling with or without pain. A history of trauma and fracture may be predated to the diagnosis. This tumor tends to be resistant to chemotherapy. We report a case of metastatic adamantinoma to the lung that responded very well to multitargeted receptor tyrosine kinase inhibitor sunitinib.

## 2. Case Presentation

**Figure 1:**
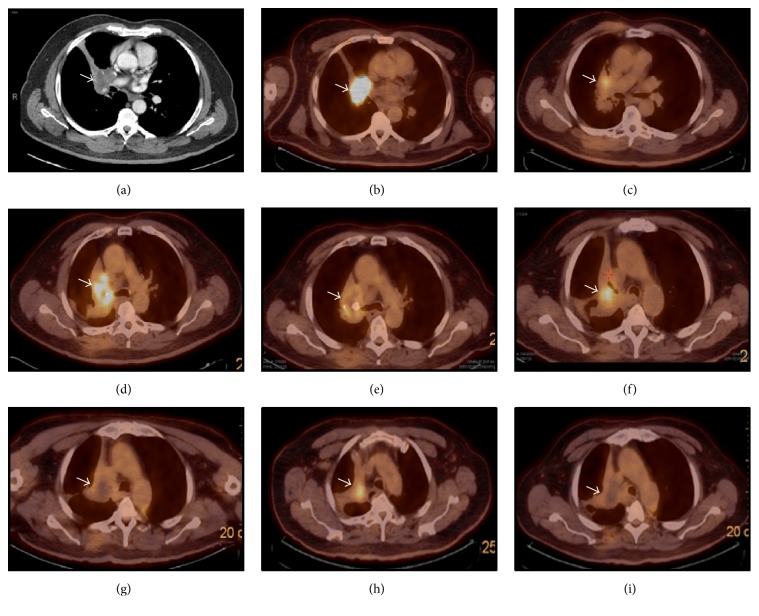
(a) CT scan showed right hilar mass (arrow) in July 2009. (b) PET scan confirmed FDG activity of the mass. (c) Partial response after radiation therapy (October 2009). (d) Extensive disease progression (May 2011). (e) Partial response after chemotherapy (December 2011). (f) Disease progression (March 2012). (g) Reduced metabolic activity after sunitinib (August 2013). (h) Disease progression (September 2014). (i) Complete disappearance of FDG activity with central necrosis after increased dose of sunitinib (February 2015).

**Figure 2:**
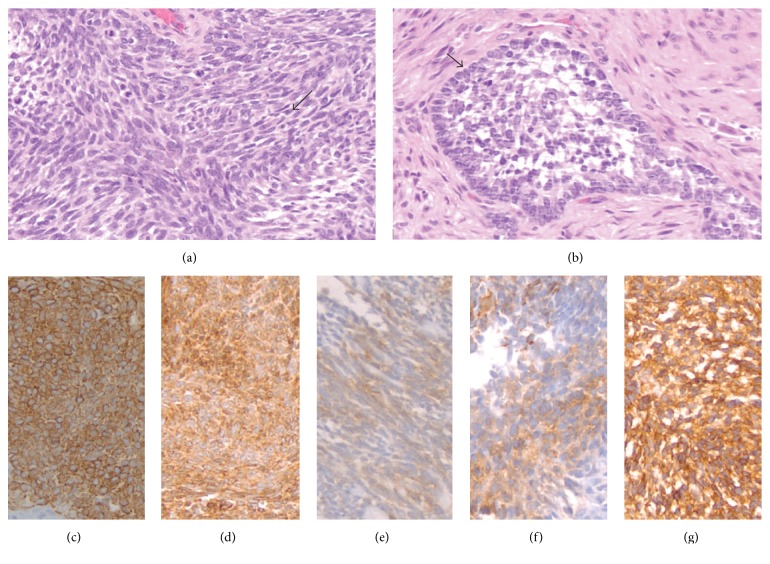
(a) Predominant spindle cell pattern; see arrow (H&E). (b) Basaloid pattern with peripheral palisading; see arrow (H&E). (c) CK5/6 strong positive. (d) BCL-2 positive. (e) CD117 focal positive. (f) VEGF focal positive. (g) EGFR diffuse positive.

A 57-year-old Caucasian male presented to Veterans Affairs Pittsburgh Healthcare System (VAPHS) with a left tibial mass in 1999. He had undergone a resection. Due to the limitation of the immunohistochemical staining at that time it was initially diagnosed as fibrosarcoma. The tumor consists predominantly of spindle neoplastic cells arranged in fascicles with occasional mitoses and stained negative with CAM5.2 and EMA. Two years later, he was found to have an enlarged left inguinal lymph node. Excisional biopsy was read as metastatic fibrosarcoma. Histologically it was similar to the original resected tumor and stained negative with AE1/AE3 and desmin and positive with vimentin. Ten years after the initial diagnosis, he presented with coughing and shortness of breath. CT scan revealed a right perihilar mass (Figure 1(a)) and PET scan showed metabolic activity in the right hilar mass (Figure 1(b)). Bronchoscopy and biopsy of the mass were consistent with metastatic adamantinoma. Histologically it was similar to the specimen that was resected from the left tibia and left inguinal lymph node. The slides from tibia and inguinal lymph node were reviewed again with additional immunohistochemical stains. The tumor has predominantly spindle cell pattern (Figure 2(a)) with focal areas of basaloid pattern showing peripheral palisading (Figure 2(b)) and fibrous stroma. Both the spindle and basaloid components are epithelial that showed immunoreactivity for epithelial markers CAM5.2, CK5/6 (Figure 2(c)), and K903. It was also positive for BCL-2 (Figure 2(d)), p63, p53, and vimentin. Immunostaining for melanoma markers (S-100 and HMB-45), vascular markers (CD34 and CD31), CK7, CK20, CD99, actin, TTF-1, and CD56 were all negative. Pankeratin and c-kit (CD117) showed focal positivity (Figure 2(e)). Ki-67 proliferation marker was approximately 35% in the epithelial component. The slides were reviewed and concurred by a bone pathologist at the University of Pittsburgh Medical Center.

He received radiation therapy to the right hilar mass for a total dose of 7500 cGy, and PET scan revealed a partial response (Figure 1(c)). Follow-up PET scan 19 months later revealed progression of disease in the right hilar mass with atelectasis of the right upper lung (Figure 1(d)) and a new small metastatic focus was detected in the left lung. The case was presented in multidisciplinary tumor board meeting. The consensus was that he needed a repeat biopsy of the right hilar mass to rule out concomitant primary lung malignancy because he was a heavy smoker. The left lung nodule was too small and difficult to approach for a biopsy. Repeat biopsy of the right hilar mass revealed persistent metastatic adamantinoma. Additional immunostaining from the biopsy showed focal positivity for VEGF (Figure 2(f)) and diffuse positivity for EGFR (Figure 2(g)). We started him on chemotherapy in June 2011. Chemotherapy consists of carboplatin with AUC of 6 on day 1 and etoposide 100 mg/m^2^ daily on days 1, 2, and 3 every 3 weeks for total of 9 cycles. PET scan in December 2011 showed partial response (Figure 1(e)) but 4 months later a surveillance PET scan showed rapid increase in size and metabolic activity of the right hilar mass (Figure 1(f)). In May 2012 we started him on sunitinib 37.5 mg orally, once daily for 4 weeks, followed by 2 weeks off, in a repeated 6-week cycle. He tolerated the drug very well except mild tiredness. He was diagnosed with hypothyroidism and was placed on levothyroxine. Serial PET scans continue to show improvement with decrease in the size of hilar mass with reduced metabolic activity (Figure 1(g)). PET scan in September 2014 showed progression in metabolic activity (Figure 1(h)). We increased the dose of sunitinib to 50 mg daily. The last PET scan in February 2015 revealed that the hilar mass developed central necrosis without metabolic activity (Figure 1(i)). The patient had continued taking of sunitinib until May 2015 when he developed hemoptysis and was admitted for pneumonia at a local hospital. He died due to complication of pneumonia at the age of 72.

## 3. Discussion

Adamantinoma is a primary low-grade, malignant bone tumor of epithelial origin. It is a rare neoplasm and comprises only 0.1–0.5% of all primary bone tumors. In 1913, Fischer named the lesion “primary adamantinoma of the tibia” because of its striking histologic resemblance to the “jaw adamantinoma” (ameloblastoma) [3]. This tumor mostly occurs in the second to fifth decades with median age of 25 to 35 years. It is more common in men than women. The tumor has a predilection in the tibia (80 to 85% of cases). In 10 to 15% of cases, the tumor is also found in the ipsilateral fibula and other major limb bones such as humerus, ulna, femur, fibula, radius, ribs, and spine as primary sites [4]. The etiology of this tumor is thought to be from displacement of basal epithelium of skin during embryological development. The theory behind predominantly tibial bone involvement by this tumor is enchondrally formed bone closest to the skin surface of tibia [5]. Adamantinoma is a tumor of epithelial origin based on immunohistochemical and ultrastructural studies. The tumor cells show strong positive staining with pan cytokeratin. In our case the tumor has positive immunoreactivity for epithelial markers CAM5.2, CK5/6, K903, and pankeratin. Ultrastructurally, the tumor cells have epithelial characteristics such as basal lamina, desmosomes, gap junctions, epithelial specific keratin, and extracellular composition similar to epithelial tissue [6].

The initial symptom of adamantinoma is swelling with or without pain involving tibia. The onset is insidious and slow but progressive in nature. Trauma has been reported in about 60% of cases. Pathological fracture may be present. Paraneoplastic manifestations, such as hypercalcemia, have been associated with tibial adamantinoma with lung metastasis. Adamantinoma is most often located in the diaphyseal area of tibia. The bone lesion is usually multilocular with sclerotic margin and overlapping with radiolucencies. These multifocal radiolucencies were surrounded by ring-shaped densities producing the characteristic “soap-bubble” appearance [2]. The differential diagnosis of adamantinoma includes osteofibrosis dysplasia (OFD) and fibrous dysplasia. OFD is located in the cortex without involvement of medullary canal while adamantinoma usually involves anterior cortical bone with extension toward the bone marrow [7].

There are two different types of adamantinoma: classic and differentiated. Classic type usually occurs in patients older than 20 years old, whereas differentiated type such as osteofibrous dysplasia- (OFD-) like adamantinoma occurs in patients younger than 20 years old. Classic type is characterized by admixture of both epithelial and osteofibrous components while OFD-like adamantinoma is predominantly osteofibrous tissue [8]. The term dedifferentiated adamantinoma means complete loss of epithelial differentiation and is usually used for tumor that is transforming to a more sarcomatoid differentiation. Immunohistochemically all adamantinomas have been uniformly positive for cytokeratins 5, 14, and 19 which represents basal epithelial cell keratins. Vimentin has been reported to be positive as well. The immunoreactivity of other bone and soft tissue tumors with known epithelial characteristics such as synovial sarcomas, epithelioid sarcomas, and chordomas has positive cytokeratins 8 and 18 [9].

Bovée et al. studied 25 cases by immunohistochemistry. The expression of fibroblast growth factor type 2 (FGF-2) and its receptor (FGFR) was present in both components of adamantinoma, but predominantly in the epithelial component. The expression of epidermal growth factor (EGF) and its receptor (EGFR) was restricted to the epithelial component. This expression was most intense and in a higher percentage of cells in classic adamantinoma. Proliferative marker Ki-67 was found exclusively in the epithelial component. During progression of adamantinoma, the epithelial cells acquire expression of FGF-2, EGF, and EGFR, accompanied by a higher proliferative activity [10].

Surgery is the main treatment for localized adamantinoma. En bloc tumor resection with wide surgical margin and limb salvage and reconstruction will likely decrease local recurrence. In a review of 70 patients, en bloc tumor resection with wide margins and limb salvage surgery was shown to have 10-year survival rate of 87.2% [11]. Amputation has not been shown to improve survival. Once adamantinoma metastasizes the prognosis is poor. This tumor has been reported to be resistant to radiation therapy and chemotherapy. Cisplatin, carboplatin, and etoposide have been reported with some response, but tumors relapse quickly [12].

Our patient has metastatic adamantinoma to the lung 10 years after the diagnosis of his primary tibial disease. The biopsy from the tibia and the inguinal lymph node metastasis were incorrectly diagnosed as fibrosarcoma due to the spindle cell predominant morphology with occasional mitoses and limited availability of immunohistochemical stains in our institution in 1999. Biopsy on the hilar mass was compared with the biopsy of inguinal lymph node and tibial mass and confirmed to have the same adamantinoma diagnosis. This diagnosis was reviewed and concurred by a bone pathologist at University of Pittsburgh Medical Center. Figures 2(a) and 2(b) showed spindle cell pattern and basaloid pattern with peripheral palisading, respectively. Czerniak et al. reported that, in the basaloid variant, the epithelial cells exhibit solid nests of basaloid cells with distinctive peripheral palisading. The spindled form shows uniform spindling with presence of clefts lined by epithelial cells [2, 7]. The epithelial component of tumor cells in our case showed immunoreactivity for all cytokeratin markers, EGFR, CD117, and VGEF. Ki-67 proliferation marker was approximately 35% in the epithelial component. We elected to start our patient on multikinase inhibitor sunitinib because of his expression of EGFR, CD117, and VGEF. We started at a lower dose of 37.5 mg orally daily for 4 weeks on 2 weeks off schedule in May 2012. The right hilar mass responded very well to therapy. Follow-up PET scan in September 2014 showed progression, so we increased the dose of sunitinib to 50 mg daily with the same schedule. The last PET scan in February 2015 revealed central necrosis and disappearance of FDG activity. He died due to complication of pneumonia in May 2015.

There are only three published case reports in the literature of using VEGFR inhibitor. Dudek et al. reported a patient with right tibial adamantinoma with lung metastasis that responded well to sunitinib 50 mg daily for 4 weeks on and 2 weeks off schedule. The lung metastasis continues to be stable for 11 months of sunitinib therapy. The tumor cells from lung biopsy in this case showed focal positive immunostaining for CD117 (c-kit) and vascular endothelial growth factor receptor-2 (VEGFR-2) and weakly positive for platelet-derived growth factor receptor beta (PDGFR-beta) [13]. Cohen et al. reported a case of metastatic adamantinoma to the lung after 4 years of primary tumor tibial resection. The patient received ifosfamide, mesna, and doxorubicin and subsequently he was treated with gemcitabine and paclitaxel. Two months later the lung metastasis progressed. He then received pazopanib, a multityrosine kinase inhibitor at 800 mg orally per day. After 6 months the lung metastasis progressed radiologically [14]. Unal et al. recently reported a case of metastatic adamantinoma. They used pazopanib and alisertib (a small molecule Aurora kinase inhibitor) with a good response for 5 months [15].

Sunitinib inhibits VEGFR, PDGFR-*β*, and c-kit. These receptors are responsible for both tumor angiogenesis and tumor cell proliferation. Inhibition of these targets leads to reduced tumor vascularization and induced cancer cell death [16]. In our case, expression of VEGF and c-kit (CD117) may explain the response of this tumor to sunitinib. We did not test VEGFR but considering the excellent response to sunitinib for 3 years the tumor could have been positive for VEGFR as well. The tumor was also focally positive for proliferation marker Ki-67 in the epithelial component. Bovée et al. reported that Ki-67 was mostly found in the epithelial component of the tumor rather than in the osteofibrous component. This epithelial component has expression of FGFR, EGFR, and Ki-67 when they metastasize to other organs just like in our case [10]. Our case is the fourth reported case of metastatic adamantinoma that responded very well to multireceptor tyrosine kinase inhibitor with the longest survival.

## 4. Conclusion

Metastatic adamantinoma has been reported to be resistant to chemotherapy. Immunohistochemistry testing of VEGFR, PDGFR, c-kit (CD117), and EGFR is paramount in this disease. Expression of those receptors in the epithelial component of the tumor has been proven to predict a good response and may prolong survival. Our patient received multikinase inhibitor sunitinib and the disease had been stable with a very good partial response for 3 years. He died of pneumonia. This is by far the longest survival for metastatic adamantinoma. We would recommend sunitinib, pazopanib, or other new multikinase inhibitors to treat this disease.
